# Heart rate variability comparison between young males after 4–6 weeks from the end of SARS-CoV-2 infection and controls

**DOI:** 10.1038/s41598-022-12844-8

**Published:** 2022-05-25

**Authors:** Mateusz Soliński, Agnieszka Pawlak, Monika Petelczyc, Teodor Buchner, Joanna Aftyka, Robert Gil, Zbigniew J. Król, Jan J. Żebrowski

**Affiliations:** 1 Faculty of Physics, Warsaw University of Technology, Warsaw, Poland; 2 Central Clinical Hospital of the Ministry of the Interior and Administration, Warsaw, Poland; 3 Mossakowski Medical Research Centre, Polish Academy of Sciences, Warsaw, Poland

**Keywords:** Nonlinear phenomena, Cardiovascular biology, Cardiology, Medical research

## Abstract

Due to the prolonged inflammatory process induced by infection of the novel severe acute respiratory syndrome coronavirus-2 (SARS-CoV-2), indices of autonomic nervous system dysfunction may persist long after viral shedding. Previous studies showed significant changes in HRV parameters in severe (including fatal) infection of SARS-CoV-2. However, few studies have comprehensively examined HRV in individuals who previously presented as asymptomatic or mildly symptomatic cases of COVID-19. In this study, we examined HRV in asymptomatic or mildly symptomatic individuals 5–7 weeks following positive confirmation of SARS-CoV-2 infection. Sixty-five ECG Holter recordings from young (mean age 22.6 ± 3.4 years), physically fit male subjects 4–6 weeks after the second negative test (considered to be the start of recovery) and twenty-six control male subjects (mean age 23.2 ± 2.9 years) were considered in the study. Night-time RR time series were extracted from ECG signals. Selected linear as well as nonlinear HRV parameters were calculated. We found significant differences in Porta’s symbolic analysis parameters V0 and V2 (*p* < 0.001), α_2_ (*p* < 0.001), very low-frequency component (VLF; *p* = 0.022) and respiratory peak (from the PRSA method; *p* = 0.012). These differences may be caused by the changes of activity of the parasympathetic autonomic nervous system as well as by the coupling of respiratory rhythm with heart rate due to an increase in pulmonary arterial vascular resistance. The results suggest that the differences with the control group in the HRV parameters, that reflect the functional state of the autonomic nervous system, are measurable after a few weeks from the beginning of the recovery even in the post-COVID group—a young and physically active population. We indicate HRV sensitive markers which may be used in long-term monitoring of patients after recovery.

## Introduction

The autonomic nervous system (ANS) has access to the deepest information about the homeostatic state of the body. Many different techniques of autonomic regulation assessment are applied in selected research settings (pupillometry, microneurography, lower body negative pressure, the cold pressor test, Ewing’s protocol, etc.), however, their use in clinical situations is usually limited^1^. This is mainly due to a lack of standardization, limited access to equipment, and the long time needed for the measurements. In effect, such measurements are difficult to obtain in a large cohort. However, having access to non-invasive data such as RR intervals (time intervals between successive R peaks in electrocardiogram), respiratory intervals or blood pressure recordings, advanced methods of signal analysis can be used for an objective assessment of the clinical condition of the patient. It has been shown that autonomic activity can be assessed by the dedicated analysis of heart rate variability (HRV)^2,3^. However, as the diagnostic method is indirect, it requires a special language of description. This language is defined by a set of computational methods, that assess such exotic properties of the signal as complexity, regularity, or scaling. Although this approach is considered esoteric and far too complex for regular clinical use, there exist certain cases, in which this unique information was shown to be useful. One of the contributions of HRV analysis is monitoring the inflammatory processes as an effect of viral infections. In the time of the Severe Acute Respiratory Syndrome Coronavirus-2 (SARS-CoV-2) pandemy, this noninvasive method is very attractive in analysis of the ANS changes over the infection time as well as during the recovery period.

The ANS modulates heart rate variability in viral infections due to the inflammation, developed by the activity of the immune system. Autonomic changes are visible in reducing HRV parameters such as standard deviation, RMSSD (root mean square of successive differences between normal heartbeats), sample entropy, multiscale entropy, power-law coefficient before sepsis in adults^4^, or in septic neonates with low birth weight – a clinical group which requires special care^5^. In the study by Mattéi et al.^6^, a decrease of the spectral indices in low (LF), high (HF), and very low (VLF) frequencies as well in the total power was observed in infants over one year of age, who have been infected with the H1N1 flu virus. La-Orkhun et al.^7^ investigated the occurrence of arrhythmias and changes in HRV in children as a result of Dengue virus infection by comparing the results of the HRV analysis in the convalescent stage and during the follow-up visit (at least 14 days after defervescence). However, in this study, there were no statistical differences of time and frequency HRV parameters calculated from 5-min RR time interval series between the study groups.

Since the beginning of the SARS-CoV-2 pandemic, several reports focusing on the HRV analysis related to the Coronavirus disease 2019 (COVID-19) infection have been published, considering either the time duration of the infection and of the recovery period^8–13^. Continuous monitoring of HRV parameters such as the standard deviation of NN (normal-to-normal) intervals (SDNN) and spectral parameters found them as markers of inflammatory processes and predictors of illness severity and mortality in critically ill patients. A change of the autonomic balance into an increasing parasympathetic tone was found in severe COVID-19 cases in comparison to mild cases or healthy subjects^9,10^. In^8^ decreasing SDNN correlated with an increase of the inflammatory marker CRP (C-reactive Protein). Differences in the autonomic responses between patients with COVID-19 in relation to patients with sepsis (due to various causes) have also been described, indicating the unique expression of a SARS-CoV-2 infection^13^. More detailed discussion about the prognostic value of HRV markers and their correlations with inflammatory markers in COVID-19 disease can be found in^9^.

To our knowledge, there is a limited number of studies on the assessment of the ANS activity in subjects encompassing asymptomatic or mild symptoms of a SARS-CoV-2 infection after a few weeks from the beginning of recovery^14^. The main research goal in our study is to investigate the HRV properties in a group of young males (20–25 years) 4–6 weeks following SARS-CoV-2 infection. Convalescents were asymptomatic or showed mild symptoms of infection and were without accompanying diseases. This group of patients is at risk for heart muscle inflammation and heart rate abnormalities. The investigation toward long term changes of ANS activity due to SARS-CoV-2 infection using noninvasive techniques may be clinically valuable. We hypothesize that HRV descriptors of ANS activity differentiate mild-symptoms convalescents of SARS-CoV-2 infection from controls. Additionally, we would like to note that such a comparison reflecting the properties of ANS may be obtained from Holter monitoring—easily used in clinical cohorts. In the research, we applied HRV markers known as the gold standard^15^ but also nonlinear parameters, among which a new one was proposed. In case of significant differences in HRV markers with a matched control group, it should be possible to select sensitive parameters that reflect the compensatory mechanisms in the circulatory system due to COVID-19 and the inflammatory process.

## Methods

### Participants

In our comparative study, we analyzed RR intervals from 24-h Holter ECG recordings measured in a group of post-COVID patients, 4–6 weeks after the second negative RT PCR (reverse transcriptase polymerase chain reaction) test for SARS-CoV-2 (the duration between the date of positive PCR test and research visit was between 5 and 7 weeks) and in a control group, without a history of infection by the virus.

Seventy physically fit male patients without comorbidities who were diagnosed with COVID-19 were enrolled in the study, all of them were recruited from the students of The Main School of Fire Service in Warsaw, Poland (April–May 2020). They suffered from the COVID-19 disease at the same time, so we decided to evaluate this group of patients, considering high clinical homogeneity and the same place of the studying; most of the students participating to this school as well as the patients with positive SARS-CoV-2 tests, recruited to our study, are male. After 4–6 weeks from the second negative PCR test for the SARS-CoV-2 virus, participants were asymptomatic (52 subjects) or showed only mild symptoms during the infection (reported symptoms: dry cough—2 subjects, dyspnea—1 subject, taste disorders—6 subjects, smell disorders—9 subjects). The mean age of these convalescents was: 22.6 ± 3.4 years. Twenty-six healthy men, mean age 23.2 ± 2.9 years, without a history of SARS-CoV-2 infection, were recruited for the control group. In the controls, the inclusion criteria were: (i) no previous confirmed infection with SARS-CoV-2 virus, (ii) age 20–25 years, (iii) male gender, (iv) no diagnosed cardiovascular diseases. The subjects in this group underwent a PCR test and an examination of the IgM and IgG levels for the SARS-CoV-2 virus for excluding active or past infection before inclusion in the study. Patients were diagnosed with COVID-19 by RT-PCR on a commercial swab test of the upper respiratory tract. The enzyme-linked immunosorbent assay (ELISA) tests were used for qualitative detection of serum anti-SARS-CoV-2 IgM, IgA and IgG antibodies (kit produced by Vircell Microbiologist, Granada, Spain: ref. MA1032 and G1032) in the study and control group.

The results of the HRV analysis are based on sixty-five recordings from the study group. Five time series were rejected due to the low quality of the signal. All sets from the control group were used in the comparison study. Baseline characteristics (expressed as mean and standard deviation), including clinical and biological parameters, are shown in Table 1. All procedures performed in studies involving human participants were in accordance with the ethical standards of the institution and with the 1964 Helsinki declaration and its later amendments. It was approved by The Local Committee of Ethics (Ethics and Supervision Committee for Research on Humans and Animals at the Central Clinical Hospital of the Ministry of Interior in Warsaw, Poland) no 89/2020. All participants gave informed written consent to participate in the study.

**Table 1:** Baseline characteristics of the study population.

Parameter	Control group	Study group (post-COVID)	*P*
n	26	65	–
age	23.2 (2.7)	22.6 (3.4)	0.134
BMI [kg/m^2^]	23.8 (2.5)	24.0 (2.0)	0.775
BSA (body surface area) [m^2^]	2.04 (0.17)	2.00 (0.13)	0.272
SBP [mmHg]	124.5 (12.4)	128.4 (12.1)	0.175
DBP [mmHg]	71.6 (10.0)	71.3 (7.5)	0.913
Interleukin 6 (ng/L)	1.83 (0.36)	2.08 (0.78)	0.202
CRP	0.76 (1.08)	0.74 (1.32)	0.491
Leucocytes (G/L)	5.59 (1.46)	5.68 (1.12)	0.443
Procalcitonin (μg/L)	0.05 (0.03)	0.06 (0.03)	0.757
Troponin I (ng/ml)	1.42 (2.56)	2.72 (4.56)	0.052
D-dimer (µg/L)	206.4 (98.3)	218.5 (186.8)	0.897
Potassium (mmol/L)	4.24 (0.32)	4.29 (0.24)	0.564
Sodium (mmol/L)	140.9 (2.21)	140.8 (1.38)	0.902

### Heart rate variability

Six hours in length, night-time RR time interval series were extracted from twenty-four hours Holter (LifeCard, Reynolds Medical) recordings and used for HRV analysis. Extraction processes were performed automatically by Holter software. Then the signals were verified and corrected by a specialist. The HRV parameters considered in the study are described below.

### Linear methods

Linear methods (both in the time domain and in the frequency domain) are the most popular and commonly used measures of HRV analysis^15,16^. In this paper, the following markers were selected for the analysis of the HRV: mean RR interval (meanRR), SDNN, root mean square successive differences between successive RR intervals (RMSSD), percentage of consecutive differences intervals greater than 50 ms (pNN50). The first metric, meanRR, is expressed in milliseconds and carries information about the average duration of a single RR interval. This measure is supported by another parameter, SDNN, which is often used as the main HRV marker. SDNN shows the scatter of RR intervals around the meanRR.

### Frequency domain

Signal analysis in the frequency domain is based on the calculation of the power spectrum determined by a Fourier Transform in the specific frequency ranges. In the spectrum of the HRV, the following frequency bands are usually examined^15^: very low (VLF; 0.003–0.04 Hz), low (LF; 0.04–0.15 Hz) and high (HF; 0.15–0.40 Hz).

### Non-linear methods

Another group of methods proposed in our study is non-linear. One representative nonlinear parameter is sample entropy^17^, used among other things as a predictor of neonatal sepsis^18^. We used the following parameters of the sample entropy: embedding dimention m = 2, tolerance r = 0.2*standard deviation of RR intervals and delay τ = 1. Another subgroup of parameters are symbolic—V0 and V2 proposed by Porta et al.^19^. In this approach, the values of the datasets are divided into N = 6 equal ranges and every range is denoted by a different symbol. Then sequences of the successive three RR intervals are collected and the RR intervals are replaced with symbols, according to the previously constructed ranges for each symbol. Every three symbols represent a symbolic word. The V0 parameter determines the percentage of words consisting of the same symbols (which is related to low variability in the time series), while the V2 parameter is equal to the percentage of words composed by three different symbols (high variability).

Other non-linear measures are derived from Detrended Fluctuation Analysis (DFA)^20^. There are two scaling exponents α_1_ and α_2_, which refer to selected time scales in HRV data. As a result, they can be used to assess the correlations in short (4–16 RR intervals) and long (16–64 RR intervals) time windows. The latter is correlated with the power of the spectrum in the LF and VLF bands^21^. Correlations were also observed between α_2_ and short-term changes in heart rhythm resulting from physiological arousals in a polysomnographic study^22,23^.

The next group of parameters was determined from the phase rectified signal averaging (PRSA) method, used for the analysis of the activity of the ANS by various clinical groups^24–27^. The PRSA method was introduced for the analysis of non-stationary and noisy data with periodicities^24^, which are often difficult to observe in the frequency domain. Therefore, the study of long signals is reduced to shorter by specific way of averaging. The first step of the method is defining the anchor points. An anchor can be every RR interval, which is longer than the previous one, which means that it reflects the ability of the system to lengthen the heart cycle, i.e., decelerate. The second step of the PRSA method is the definition of the radius. Usually, the radius parameter is strictly related to the frequency scale of the periodicities obtained around the anchor points. Slow oscillations require a larger radius than fast ones. In our study, we used 1024 points. Such length was chosen as satisfactory for Fast Fourier Transformation (FFT) and enough for the observation of respiratory oscillations. The window length is twice the radius long. There are RR intervals preceding the anchor points and as well following it. Windows are aligned in such a way that the anchor points coincide. The final PRSA curve is obtained by averaging the RR intervals over the aligned windows.

In our paper, we expanded the analysis of the PRSA curve to explore the respiratory oscillations as seen through RR intervals. Following the idea given in^24^, we used the PRSA curve as an input signal for the FFT. The averaged PRSA signal shows the properties of the power spectrum of the RR intervals (such as the characteristic frequencies), which can be recognized more clearly than for the original HRV data^24^. So far, the PRSA technique has been used to analyze the parameters of the averaged curve. For this purpose, diagnostic markers DC (decelaration capacity) and AC (acceleration capacity) were introduced^24,25^. The DC parameter was used as a predictor of mortality in patients after myocardial infarction^25^. In the FFT technique applied to the PRSA curve, the sampling frequency is the reciprocal of the mean RR interval. As a result, the frequency representation is not given in Hz but as a multiplicity of the reciprocal of the mean RR interval. In our study, we propose to determine the region in the power spectrum, which corresponds to the HF range. Then, we detected its maximum position (denoted as Resp. Peak). The Resp. Peak is a marker, which specifies the number of averaged RR intervals per respiratory period. It can be easily used for assessing the coupling between heart rhythm and respiration^28,29^. We also propose a parameter, which estimates the width of the respiratory peak (Resp. range). The wider the range, the larger variability of the respiratory period. Both proposed markers characterize respiration oscillations that manifest themselves in HRV data.

### Statistical analysis

Comparison of the mean values of HRV parameters (as well as the baseline characteristic parameters in Table 1) between the study and the control group was undertaken using the parametric Student’s t-test for independent groups or the nonparametric Mann–Whitney test if the distribution were not normal (tested by Shapiro–Wilk test); *p* < 0.05 was considered as significant. Statistical analyses were performed using the R package (ver. 3.6.0) with R-Studio software (ver. 1.2.1335).

## Results

The quantitative results are presented as X(Y), where X is the mean and Y standard deviation. Table 2 showed the results for the linear, nonlinear, and PRSA parameters, respectively. Significant differences were found for the VLF range (*p* = 0.02), symbolic analysis parameters: V0 and V2 (both *p* < 0.001), α_2_ (*p* < 0.001), and Resp. Peak (*p* = 0.012) parameter from PRSA method.

**Table 2:** Comparison of the mean HRV parameters between the study groups.

Parameter	Control group (n = 26)	Study group (post-COVID) (n = 65)	*p*
**Linear parameters in the time domain**			
Mean RR [ms]	1115 (122)	1162 (111)	0.093
SDNN [ms]	129 (30)	139 (33)	0.166
RMSSD [ms]	91 (35)	90 (39)	0.857
pNN50 [%]	41.9 (17.4)	47.8 (18.4)	0.109
**Frequency domain**			
VLF [ms^2^]	10,490 (4762)	13,826 (6600)	0.022
LF [ms^2^]	2879 (1471)	2889 (1606)	0.742
HF [ms^2^]	3084 (2704)	3063 (2665)	0.816
**Non-linear analysis**			
V0 [%]	66.5 (18.2)	49.0 (15.4)	< 0.001
V2 [%]	7.5 (6.3)	14.7 (6.9)	< 0.001
Sample entropy	1.27 (0.20)	1.32 (0.18)	0.357
α_1_	0.99 (0.15)	1.05 (0.16)	0.082
α_2_	1.03 (0.10)	1.11 (0.08)	< 0.001
**PRSA**			
DC [a.u.*]	26.8 (10.1)	26.6 (9.9)	0.878
AC [a.u.]	− 23.9 (7.0)	− 23.1 (7.5)	0.816
Resp. peak [a.u.]	4.25 (0.61)	3.91 (0.56)	0.012
Resp. range [a.u.]	0.281 (0.031)	0.275 (0.030)	0.425

*a.u.- arbitrary units, see details in the “Methods” chapter.

## Discussion

The study indicates significant differences in HRV parameters between the convalescents and the control group, which support the hypothesis that a SARS-CoV-2 infection influences heart rate variability in the recovery period. Significant differences were observed mostly for non-linear parameters which had not been analyzed and reported in the previous studies. Non-linear methods refer to the “complexity” of the RR series. Complexity is related to the irregularity of RR intervals in the records. In the extreme case, when the same value of the RR time interval (the ideal case of a metronomic pacemaker activity) is present, there is a lack of complexity.

The V0 and V2 from Porta’s symbolic method were among the non-linear paratmeters of HRV that showed statistically significant differences between the properties of the goups studied here. The value of V0 was lower, and the value of V2 was higher for convalescents. Following the interpretation proposed by Guzzetti et al.^30^, such representation may be explained as an increase in parasympathetic cardiac modulation. The interpretation of V2 related to the parasympathetic activity seems quite probable if one recalls that the parasympathetic branch has the potential to introduce fast (beat to beat) changes in the heart rate, which may even lead to an asystole. The effect of autonomic dysfunction in the early stage of COVID-19 was observed previously in patients with different intensities of symptoms^31^ as well as in critical conditions^9^. In the Maartje et al. study, the authors discussed the separate roles of the sympathetic branch and vagus nerve in the infection^12^. The vagus nerve seems to inform the brain about a peripheral inflammation. As a result, the production of cortisol (causing suppressing the inflammation) is activated through the hypothalamic–pituitary–adrenal axis. The Authors also pointed out that due to the descending efferent vagal-to-sympathetic neural conversion, a subclass of T-cells secrete acetylcholine and the production of inflammatory cytokines by splenic macrophages is limited. When the vagus activity is low, then the inflammatory response can even lead to a ‘cytokine storm’. However, in physiological response, the vagus ‘controls’ the inflammation process.

Another result is the occurrence of a significant difference in the Resp. Peak parameter, related to the coupling of respiratory rhythm to the heart rate. The Buchner et al.^28^ study proved the significance of this parameter (more specifically of the equivalent parameter: mean rhythm ratio) in the analysis of this coupling, showing an increase in this parameter from values 3–4 to 8 after a complete pharmacological blockade of the ANS. The role of such coupling is to maintain homeostasis (perhaps a better term here would be: allostasis) between ventilation and perfusion^32^. The differences in respiratory rhythm between the study and the control group might be related to the long-term effects of the SARS-CoV-2 virus on the respiratory system, for example, due to a reduction in tidal volume^33^, or to an increase in pulmonary arterial vascular resistance, which is observed in patients after COVID-19 even with mild symptoms of the disease^34^. The modulation of the cardiovascular coupling, resulting from adaptation processes during the convalescence period and mediated by the ANS, may be the primary factor affecting those HRV parameters for which we observed significant differences with the control group. It is interesting, that Resp. Peak parameter, suitable for noninvasive screening (e.g., using modern heart rhythm monitors, which are very popular nowadays) allows us to observe changes, which may be considered a manifestation of the possible increase of vascular resistance. This primary observation provides an open area for a subsequent pilot study.

Our results suggesting an increase of the parasympathetic nervous system activity seems to be at least semi-contrasting with Stute et al. study^14^ where increasing of sympathetic activity was observed (by measuring muscle sympathetic nerve activity, MSNA). However, in our opinion there is no contradiction between pronounced respiratory modulation and the possibility of an increased level of sympathetic activity, as the frequency ranges of those two control loops are different. The V2 parameter from Porta’s symbolic analysis, looks at local heart rate variability expressed in consecutive heart beats. It is questionable to what extent such a dynamics is altered by increased sympathetic activity. Typically, when sympathetic activity is increased, the overall variability decreases, as SDNN has a negative correlation to the mean rate. Note, however, that the Porta parameter is symbolic, which makes it insensitive on the amplitude of changes, only to the fact of changes. Moreover, the change in cardiorespiratory coupling we suggest, altered due to a suspected pathological state in pulmonary circulation, might be related to the sympathetic and parasympathetic tone in a very complex way^35–38^. Thus, we claim that the parasympathetic activity is increased, which does not mean that the sympathetic activity must not be also increased. Note that the HRV analysis results presented in the Stute et al. study, in contrast to the results obtained with MSNA also indicated an increase in parasympathetic activity.

The results did not show statistical differences for LF and HF spectral parameteras, contrary to the reference studies^9–11^, but a statistically significant difference was observed for the VLF range. The studies we cited in which a statistical significance occurred for the LF and HF parameters analyzed shorter time series (on the order of several minutes) than those analyzed in our study. For signals of several hours duration, the level of stationarity is low (lower than in shorter signals of several minutes duration), which may be an additional factor contributing to the lack of significance for the LF and HF parameters. Nonlinear parameters such as the symbolic parameters V0 and V2, for which statistical significance was obtained, depend less on the level of stationarity of the signal. Thus, they may provide a more sensitive tool in distinguishing between the test and control groups in our study. Classically, according to the HRV Task Force^15^, the LF range is mainly associated with the activity of the sympathetic component of the ANS, while the power in the HF band describes the activity of the parasympathetic system and the influence of breathing^39^. However, the frequency bands cannot be completely assigned to specific parts of the ANS and this assignment has raised many objections^40–42^. The interpretation of the VLF component modulation is not completely clear. Traditionally it is associated with thermoregulation and/or humoral regulation^43,44^ and more recently it was shown to correlate with the occurrence of the U-shaped patterns^22,23^ that have a form of infrequent events in the heart rate rather than a constant modulating frequency.

The differences of VLF and α_2_ between the groups studied may be associated with the different number of U-shaped patterns, characteristic changes in RR time interval values, lasting approximately 20–40 s, occurring during sleep^22,23^. U-shaped patterns are a common phenomenon in humans, occurring mostly during sleep and appear to be a cardiovascular response to cortical arousal. Cortical arousal is a physiological adaptive mechanism for external or internal stimuli that disrupts the state of homeostasis^23^. The study by Soliński et al.^22^ demonstrated the relation between the number of U-shaped patterns detected in night-time RR time interval series and the values of HRV parameters related to its long-time correlation, multifractal properties, and very low-frequency component. Referring to the methodology used in that report, we compared the mean values of U-shaped patterns between the study and the control group obtaining a significant statistical difference (study group: 17.1 (95% CI 15.6–18.5) patterns vs. control group 12.9 (95% CI 10.9–14.8) patterns, *p* < 0.001). The U-shaped patterns were then artificially removed from the RR time series of the groups studied here and the mean values of the HRV parameters used in the data analysis were again compared. Re-analysis comparing HRV parameters between groups showed non-significance for VLF, α_2_ parameters (*p* = 0.062 and 0.092, respectively), however, significance for V0 and V2 parameters persisted (*p* < 0.001). It is difficult to determine whether differences in the number of U-shaped patterns could be related to SARS-CoV-2 virus infection. Additional polysomnography studies should be performed to investigate possible differences in sleep dynamics between groups. In some recent studies sleep disturbances during infection were observed, even in mild COVID-19 patients^45–47^ may have implications for the results of HRV analyses. Changes in sleep dynamics during infection could also affect the number of arousals during sleep, as well as the number of U-shaped patterns.

One strength of the conducted study, taking into account the limited number of the subjects, is the selection of a fairly homogeneous populations for comparative analysis of HRV parameters (population of males in a narrow age range). The results of the study may be an input to create more complex indexes and methods such as multiparameters or artificial intelligent algorithms to diagnose autonomic dysregulation which use either HRV measures or other physiological signals, similar to the index described in^48^. Moreover, the data were obtained from Holter recordings, so physiological activity of the ANS can be observed in natural conditions, which makes the method suitable for screening and remote monitoring in a telemedicine context.

One of the clinical endpoints of the present study could be the development of a methodology for monitoring post-COVID patients, including the analysis of HRV performed by Holter monitoring. In order to validate the application of HRV measures, we suggest performing studies in groups of patients affected by COVID-19 several months after infection. In our report, the study group was characterized by a mild (or asymptomatic) course of the disease, which may indicate that the statistically significant HRV parameters are sensitive markers for a long-term monitoring of convalescent patients.

### Limitations

This study has potential limitations. It should be pointed out that HRV analysis is an indirect approach for monitoring the ANS. Its main advantage is non-invasive form, however, the interpretation of the results should be done carefully in context to its limitations^39–41^. RR time interval series were measured in uncontrolled conditions, according to a standard Holter protocol, thus the results may have been affected to some extent by unknown external factors. In addition, we did not measure Holter recordings in the study group before SARS-CoV-2 infection, so that a direct comparison of HRV analysis results before and after the advent of COVID-19 was not possible. However, repetition of the Holter measurements after a relatively long time from the infection (e.g., 1–2 years) can provide new information about the study group.

The participants were diagnosed with COVID-19 by RT-PCR on a swab test of the upper respiratory tract which can return, as every test, some false positive or false negative results. Determining the clinically false negative rate is difficult and the clinical presentation is varied and so no combination of symptoms can reliably diagnose COVID-19 infection. Radiological findings on chest X-ray and CT scans can be indicative but in many instances are not sufficient to conclusively rule in or rule out COVID-19. While RT-PCR is highly specific and remains the principal method for detecting COVID-19 infection across the world (sensitivity about 90%), understanding the false negative rate is important so that clinicians have an estimate of the reliability of the test when making management plans based on the results.

## Conclusions

The study showed significant differences in HRV parameters related to higher parasympathetic nervous system activity in post-COVID male subjects with mild symptoms in comparison to the control group who did not have COVID-19. We also observed differences between the study and the control group in regard to the characteristic frequency of the coupling between cardiac and respiratory rhythm based on the Resp. Peak parameter. We hypothesize, that these differences might be caused by the increased peripheral resistance in pulmonary circulation due to SARS-CoV-2.

The results suggest that even in the group of relatively young and physically active population an infection of SARS-CoV-2 virus may measurably influence the ANS and the cardio-respiratory system after a few weeks from the beginning of recovery. Although we now know much more about the physiological changes caused by COVID-19, the topic of long-term effects after the infection is still open. The evaluation of the degree of cardiopulmonary coupling and hypotheses about its use to assess the effects of Covid-19 is a new voice in the debate.

## References

[1] Cheshire WP (2021). Electrodiagnostic assessment of the autonomic nervous system: A consensus statement endorsed by the American Autonomic Society, American Academy of Neurology, and the International Federation of Clinical Neurophysiology. Clin. Neurophysiol..

[2] Sato Y, Ichihashi K, Kikuchi Y, Shiraishi H, Momoi MY (2007). Autonomic function in adolescents with orthostatic dysregulation measured by heart rate variability. Hypertens. Res..

[3] Ghiasi S, Greco A, Barbieri R, Scilingo EP, Valenza G (2020). Assessing autonomic function from electrodermal activity and heart rate variability during cold-pressor test and emotional challenge. Sci. Rep..

[4] Ahmad S (2009). Continuous multi-parameter heart rate variability analysis heralds onset of sepsis in adults. PLoS ONE.

[5] Griffin MP, Moorman JR (2001). Toward the early diagnosis of neonatal sepsis and sepsis-like illness using novel heart rate analysis. Pediatrics.

[6] Mattéi J (2011). Autonomic dysfunction in 2009 pandemic influenza A (H1N1) virus-related infection: A pediatric comparative study. Auton. Neurosci..

[7] La-Orkhun V, Supachokchaiwattana P, Lertsapcharoen P, Khongphatthanayothin A (2011). Spectrum of cardiac rhythm abnormalities and heart rate variability during the convalescent stage of dengue virus infection: A Holter study. Ann. Trop. Paediatr..

[8] Hasty F, García G, Dávila H, Wittels SH, Hendricks S, Chong S (2021). Heart rate variability as a possible predictive marker for acute inflammatory response in COVID-19 patients. Mil. Med..

[9] Aragón-Benedí C (2021). Is the heart rate variability monitoring using the analgesia nociception index a predictor of illness severity and mortality in critically ill patients with COVID-19? A pilot study. PLoS ONE.

[10] Kaliyaperumal D, Karthikeyan RK, Alagesan M, Ramalingam S (2021). Characterization of cardiac autonomic function in COVID-19 using heart rate variability: A hospital based preliminary observational study. J. Basic Clin. Physiol. Pharmacol..

[11] Buchhorn R, Baumann C, Willaschek C (2020). Heart rate variability in a patient with coronavirus disease 2019. Int. Cardiovasc. Forum J..

[12] Maartje BAM (2021). Heart-rate-variability (HRV), predicts outcomes in COVID-19. PLoS ONE.

[13] Kamaleswaran, R. *et al*. Changes in non-linear and time-domain heart rate variability indices between critically ill COVID-19 and all-cause sepsis patients-a retrospective study. *medRxiv*. Preprint at https://europepmc.org/article/ppr/ppr172638. 10.1101/2020.06.05.20123752 (2020).

[14] Stute NL, Stickford JL, Province VM, Augenreich MA, Ratchford SM, Stickford ASL (2021). COVID-19 is getting on our nerves: Sympathetic neural activity and haemodynamics in young adults recovering from SARS-CoV-2. J. Physiol..

[15] Task Force of the European Society of Cardiology the North American Society of Pacing Electrophysiology (1996). Heart rate variability. Standards of measurements, physiological interpretation, and clinical use. Circulation.

[16] Acharya UR, Joseph KP, Kannathal N, Lim CM, Suri JS (2006). Heart rate variability: A review. Med. Biol. Eng. Comput..

[17] Richman JS, Moorman JR (2000). Physiological time-series analysis using approximate entropy and sample entropy. Am. J. Physiol. Heart Circ..

[18] Lake DE, Richman JS, Griffin MP, Moorman JR (2002). Sample entropy analysis of neonatal heart rate variability. Am. J. Physiol. Regul. Integr. Comp. Physiol..

[19] Porta A, Guzzetti S, Montano N, Furlan R, Pagani M, Malliani A, Cerutti S (2001). Entropy, entropy rate, and pattern classification as tools to typify complexity in short heart period variability series. IEEE Trans. Biomed. Eng..

[20] Peng CK, Havlin S, Stanley HE, Goldberger AL (1995). Quantification of scaling exponents and crossover phenomena in nonstationary heartbeat time series. Chaos.

[21] Willson K, Francis DP, Wensel R, Coats AJ, Parker KH (2002). Relationship between detrended fluctuation analysis and spectral analysis of heart-rate variability. Physiol. Meas..

[22] Soliński M, Kuklik P, Gierałtowski J, Baranowski R, Graff B, Żebrowski J (2020). The effect of persistent U-shaped patterns in RR night-time series on the heart rate variability complexity in healthy humans. Physiol. Meas..

[23] Soliński M, Kuklik P, Gierałtowski J, Baranowski R, Graff B, Żebrowski J (2021). Reply to comment on ‘The effect of persistent U-shaped patterns in RR night-time series on the heart rate variability complexity in healthy humans. Physiol. Meas..

[24] Bauer A, Kantelhardt JW, Bunde A, Barthel P, Schneider R, Malik M, Schmidt G (2006). Phase-rectified signal averaging detects quasi-periodicities in non-stationary data. Physica A.

[25] Bauer A (2006). Deceleration capacity of heart rate as a predictor of mortality after myocardial infarction: Cohort study. Lancet.

[26] Hu W (2016). Deceleration and acceleration capacities of heart rate associated with heart failure with high discriminating performance. Sci. Rep..

[27] Xu YH, Wang XD, Yang JJ, Zhou L, Pan YC (2016). Changes of deceleration and acceleration capacity of heart rate in patients with acute hemispheric ischemic stroke. Clin. Interv. Aging..

[28] Buchner T (2009). On the nature of heart rate variability in a breathing normal subject: a stochastic process analysis. Chaos.

[29] Sobiech T, Buchner T, Krzesiński P, Gielerak G (2017). Cardiorespiratory coupling in young healthy subjects. Physiol. Meas..

[30] Guzzetti S (2005). Symbolic dynamics of heart rate variability: A probe to investigate cardiac autonomic modulation. Circulation.

[31] Milovanovic B (2021). Assessment of autonomic nervous system dysfunction in the early phase of infection with SARS-CoV-2 virus. Front. Neurosci..

[32] Pocock G, Richards CD, Richards DA (2017). Human Physiology.

[33] Huang Y (2020). Impact of coronavirus disease 2019 on pulmonary function in early convalescence phase. Respir. Res..

[34] Tudoran C (2021). Evidence of pulmonary hypertension after SARS-CoV-2 infection in subjects without previous significant cardiovascular pathology. J. Clin. Med..

[35] Krause H, Kraemer JF, Penzel T, Kurths J, Wessel N (2017). On the difference of cardiorespiratory synchronisation and coordination. Chaos.

[36] Narkiewicz K, Van De Borne P, Montano N, Hering D, Kara T, Somers VK (2006). Sympathetic neural outflow and chemoreflex sensitivity are related to spontaneous breathing rate in normal men. Hypertension.

[37] Smith JC, Abdala AP, Rybak IA, Paton JF (2009). Structural and functional architecture of respiratory networks in the mammalian brainstem. Philos. Trans. R. Soc. Lond. B Biol. Sci..

[38] Anrep G, Pascual W, Rössler R (1936). Respiratory variations of the heart rate-I—The reflex mechanism of the respiratory arrhythmia. Proc. R. Soc. B.

[39] Acharya UR, Joseph KP, Kannathal N, Min LC, Suri JS, Acharya UR, Suri JS, Spaan JAE, Krishnan SM (2007). Heart rate variability. Advances in Cardiac Signal Processing.

[40] Champéroux P, Fesler P, Judé S, Richard S, Le Guennec JY, Thireau J (2018). High frequency autonomic modulation: A new model for analysis of autonomic cardiac control. Br. J. Pharmacol..

[41] Billman GE (2013). The LF/HF ratio does not accurately measure cardiac sympatho-vagal balance. Front. Physiol..

[42] Eckberg DL (1997). Sympathovagal balance: A critical appraisal. Circulation.

[43] Togo F, Kiyono K, Struzik ZR, Yamamoto Y (2005). Unique very low-frequency heart rate variability during deep sleep in humans. IEEE Trans. Biomed. Eng..

[44] Tripathi KK (2011). Very low frequency oscillations in the power spectra of heart rate variability during dry supine immersion and exposure to non-hypoxic hypobaria. Physiol. Meas..

[45] Lin YN (2021). Burden of sleep disturbance during COVID-19 pandemic: A systematic review. Nat. Sci. Sleep.

[46] Alimoradi Z (2021). Sleep problems during COVID-19 pandemic and its’ association to psychological distress: A systematic review and meta-analysis. EClinicalMedicine.

[47] Bhat S, Chorkoverty S (2021). Sleep disorders and COVID-19. Sleep Med..

[48] Barizien N, Le Guen M, Russel S, Touche P, Huang F, Vallée A (2021). Clinical characterization of dysautonomia in long COVID-19 patients. Sci. Rep..

